# Determination of Non-Invasive Biomarkers for the Assessment of Fibrosis, Steatosis and Hepatic Iron Overload by MR Image Analysis. A Pilot Study

**DOI:** 10.3390/diagnostics11071178

**Published:** 2021-06-29

**Authors:** Alba Meneses, José Manuel Santabárbara, Juan Antonio Romero, Roberto Aliaga, Alicia María Maceira, David Moratal

**Affiliations:** 1 Center for Biomaterials and Tissue Engineering, Universitat Politècnica de València, 46022 Valencia, Spain; mfelipealba@gmail.com; 2 ASCIRES Grupo Biomédico, 46004 Valencia, Spain; jmsantabarbarag@ascires.com (J.M.S.); jaromerom@ascires.com (J.A.R.); raliaga@eresa.com (R.A.); amaceira@ascires.com (A.M.M.)

**Keywords:** biomarker, magnetic resonance protocol, fibrosis, steatosis, hepatic iron overload, phantom, image analysis

## Abstract

The reference diagnostic test of fibrosis, steatosis, and hepatic iron overload is liver biopsy, a clear invasive procedure. The main objective of this work was to propose HSA, or human serum albumin, as a biomarker for the assessment of fibrosis and to study non-invasive biomarkers for the assessment of steatosis and hepatic iron overload by means of an MR image acquisition protocol. It was performed on a set of eight subjects to determine fibrosis, steatosis, and hepatic iron overload with four different MRI sequences. We calibrated longitudinal relaxation times (*T1* [ms]) with seven human serum albumin (HSA [%]) phantoms, and we studied the relationship between them as this protein is synthesized by the liver, and its concentration decreases in advanced fibrosis. Steatosis was calculated by means of the fat fraction (*FF* [%]) between fat and water liver signals in “fat-only images” (the subtraction of *in-phase* [IP] images and *out-of-phase* [OOP] images) and in “water-only images” (the addition of IP and OOP images). Liver iron concentration (*LIC* [µmol/g]) was obtained by the transverse relaxation time (T2* [ms]) using Gandon’s method with multiple echo times (*TE*) in T2-weighted IP and OOP images. The preliminary results showed that there is an inverse relationship (r = −0.9662) between the *T1* relaxation times (ms) and HSA concentrations (%). Steatosis was determined with *FF* > 6.4% and when the liver signal was greater than the paravertebral muscles signal, and thus, the liver appeared hyperintense in fat-only images. Hepatic iron overload was detected with *LIC* > 36 µmol/g, and in these cases, the liver signal was smaller than the paravertebral muscles signal, and thus, the liver behaved as hypointense in IP images.

## 1. Introduction

Liver diseases have an annual mortality rate of 2 million people globally [1]. Liver fibrosis and steatosis are some of the most common hepatic diseases that consist of the accumulation of connective tissue and fat in the liver, respectively. These two liver diseases are related to non-alcoholic fatty liver disease (NAFLD) and alcoholic fatty liver disease (ALD), whose most common risk factors are obesity, diabetes, and alcohol consumption [2]. Both diseases can evolve to steatosis, non-alcoholic steatohepatitis (NASH) with or without liver fibrosis, cirrhosis, and end in hepatocarcinoma, which are the 11th and 16th leading causes of death in the world [1,3]. Another common liver disease is hepatic iron overload, or hemochromatosis, which is mainly genetic in origin and if it is not treated in time it can progress to other liver diseases, such as cirrhosis [4].

The early diagnosis of fibrosis, steatosis, and iron overload is key and fundamental to slow down the progression of the disease, its subsequent chronicity, and irreversibility because these three asymptomatic diseases are often diagnosed accidentally or in advanced stages of the disease [5].

Liver biopsy is the gold standard in diagnostic and prognosis. It is characterized mainly by its invasiveness through percutaneous extraction of a small sample of the liver that usually causes complications and adverse effects in the patient [6]. In addition, the study of the sample by pathological anatomy has sampling errors, inter- and intra-observer variability, and it does not provide information about the distribution of the disease in the liver [7].

On the other hand, non-invasive diagnostic tests are mainly based on different medical imaging modalities, such as computed tomography (CT) [8], abdominal ultrasound [9,10], magnetic resonance imaging (MRI) [11,12,13], and ultrasound and magnetic resonance elastography [14,15,16]. The cutting edge of non-invasive diagnosis of these liver diseases is focused on multiparametric ultrasound for the assessment of liver structure, fibrosis, steatosis, and viscoelastic properties in patients with NAFLD [17,18], as well as the use of two-dimensional shear wave elastography and attenuation imaging for the accurate assessment of fibrosis and steatosis in patients with suspected NASH [19].

The research line, called by some authors the *virtual biopsy* [20], is focused on quantitative diagnosis using non-invasive biomarkers on MRI. There exist many methods for dealing with the assessment of steatosis and hepatic iron overload, but few studies have focused on liver fibrosis on MRI. For this reason, the aim of this work was to study the biomarkers and the regions of interest (ROIs) for the assessment of fibrosis, steatosis, and hepatic iron overload by means of MR image analysis. We examined some previous work for steatosis and hepatic iron overload in 1.5T and 3T and proposed the protein human serum albumin (HSA) as a possible biomarker of fibrosis on an MRI protocol.

## 2. Materials and Methods

### 2.1. Phantoms

**Figure 1:**
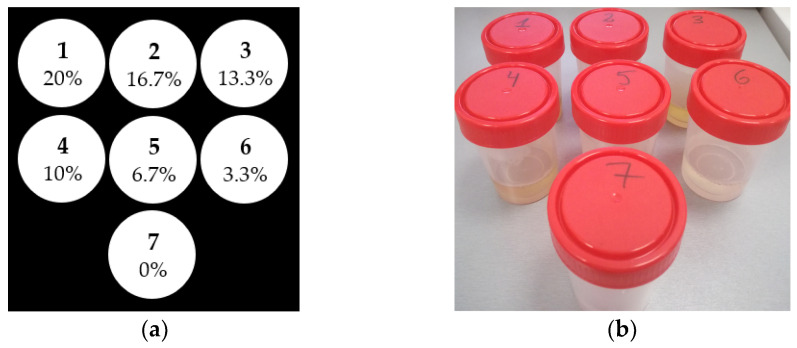
Scheme with the HSA concentrations (**a**) and photography of the 7 phantoms (**b**).

MRI phantoms were used for the calibration of the MRI relaxation times of the MRI scanner at 3T. There were 7 phantoms prepared using HSA (20% solution in 100 mL) and physiological serum at room temperature. Each phantom had a volume of 30 mL and a concentration of HSA ranged from 0 to 20%. Figure 1 shows the phantoms and their concentrations.

One of the reasons for selecting the HSA protein is because the MRI relaxation time of this protein is similar to the MRI relaxation times of the human tissue [21].

### 2.2. Subjects and Image Acquisition

The MRI acquisition protocol was applied to 8 patients (7 male and 1 female —patient #8—) with an age of 45 ± 15.2 years old (mean ± standard deviation). Table 1 presents 2 patients for the study of fibrosis (patient #1 * and #2; 41 years old for both patients), 6 patients for steatosis (patient #3 **, #4, #5, #6, #7 and #8; 46.3 ± 17.4 years old), and 5 patients for hepatic iron overload (patient #3 **, #4, #5, #6 and #7; 50.8 ± 15.5 years old).

The MRI sequences were performed on a 3T MAGNETOM Vida MRI scanner (Siemens, Erlangen, Germany) and 1.5T MRI scanner (Siemens, Erlangen, Germany). The MRI sequences for fibrosis were *B1Map_for_T1mapping* and *T1_Images_B1corr*, which is a reconstruction of the magnetic field heterogeneity of the first sequence *B1Map_for_T1mapping* [22], for steatosis was *T1_VIBE_Dixon* and for hepatic iron overload was *T2_Multi-echo*. The different MRI sequences and their parameters are described in Table 2.

The coils required for the MRI protocol were: the radiofrequency (RF) coil of the MRI scanner tunnel (*Body*) which was the transmission antenna for all the sequences and the reception antenna only for the hepatic iron overload sequence, and the anterior (*Body_30*) and posterior (*Spine_32_RS*) surface RF coils, which were the reception antennas for fibrosis and steatosis sequences.

### 2.3. Software

The software tool used to select the different ROIs for fibrosis and steatosis was *RadiAnt DICOM Viewer* (Medixant, Poznan, Poland) and for the quantification of hepatic iron overload was *MRQuantif* (Prof. Yves Gandon, University of Rennes, Rennes, France), which is available at https://imagemed.univ-rennes1.fr/en (accessed on 12 May 2021). Moreover, *Matlab R2019b* (The MathWorks, Inc., Natick, MA, USA) was used for graphic representations.

### 2.4. Regions of Interest

The different parameters, such as the values of the relaxation times or the signal intensities, were measured with several ROIs with elliptical shape. We selected 3 ROIs for the liver in the Couinaud’s segments (IV, VIII, and VII) [23], 2 ROIs for the right and left paravertebral muscles, 1 ROI for the spleen, 1 ROI for the abdominal fat, and 1 ROI for the background noise. All ROIs have to be drawn avoiding liver vessels or artifacts that modify the measures.

### 2.5. Biomarkers

#### 2.5.1. Fibrosis

Liver fibrosis consists of the accumulation of proteins in the extracellular matrix, which is synthesized by Ito cells, known as hepatic lipocytes, which produce the replacement of liver tissue by scar tissue [24]. Previous studies of liver and cardiac fibrosis demonstrated the relationship between the collagen protein concentrations and myocardial *T1* maps on MRI [20,25].

In the case of liver fibrosis, the target protein was HSA, which is synthesized exclusively by the liver. Some studies showed the importance of this protein as a significant predictor that decreases its production and concentration (60–80%) in cases of advanced fibrosis [26]. For this reason, the purpose of studying this biomarker was to know the relationship between HSA concentrations (%) and liver *T1* values (ms).

#### 2.5.2. Steatosis

Steatosis consists of the accumulation of lipids, commonly triglycerides, in the liver when it exceeds 6% of fat in hepatocytes [3,27]. Therefore, the Dixon method was used for the calculation of the fat fraction (FF), as the precession frequency of the water (W) protons is greater than the precession frequency of the fat (F) protons [28].

The transverse magnetization vectors ($\vec{M_{x,y}}$) of W and F protons with the excitation pulse are in phase but, as they have different precession frequencies, after that pulse, these vectors are out of phase until they reach the opposite phase. Therefore, it is possible to obtain in-phase (IP) and out-of-phase (OOP) images as the addition and subtraction of the signals from the protons of water and fat. In addition, fat-only images and water-only images are obtained as the subtraction and addition of the IP and OOP images respectively.

The *FF* (%) was calculated by Equation (1) with the fat signal (*S_F_*) and water signal (*S_W_*) of the liver in the fat-only and water-only images respectively [29].

*FF* (%) = (*S_F_*/*S_F_* + *S_W_*) × 100 (1)

There are different grades of steatosis depending on the gravity of the disease [27]: no steatosis (<5% of hepatocytes affected), mild steatosis (5–33% of hepatocytes affected), moderate steatosis (33–66% of hepatocytes affected), and severe steatosis (>66% of hepatocytes affected). The reference values for *FF* (%), based on Tang et al. [27], are: FF < 6.4% (no steatosis), *FF*: 6.4–17.4% (mild steatosis), *FF*: 17.4–22.1% (moderate steatosis), and FF > 22.1% (severe steatosis).

The Signal-to-Intensity-Ratio (SIR) was another method used to study the ratio between the liver signal (*S_Liver_*) and the paravertebral muscles signal (*S_Paravertebral muscles_*), which is the reference signal, as it does not change in patients with steatosis.

*SIR = S_Liver_/S_Paravertebral muscles_* (2)

#### 2.5.3. Hepatic Iron Overload

Hepatic iron overload occurs when there is an excess of accumulated iron in the liver that the human system cannot excrete [4]. The iron ions are paramagnetic substances that decrease the T2* signal and allow the diagnosis of hepatic iron overload with precision [30]. 

The calculation of T2* (ms) was obtained by the exponential model of the transverse relaxation component of the *S_Liver_* as a function of *TE* (ms) according to Equation (3) [31].

*S_Liver_ (TE) = S_Liver_* (0) · exp (−TE/T2*) + Noise (3)

The study of Gandon et al. [32] established a relationship between the results of liver biopsy and the results of 1.5T MRI scanner in a patient sample. In addition, it is possible to calculate liver iron concentration (LIC) at 3T with Equation (4), as T2* is approximately half at 3T than at 1.5T [32]:

*LIC* (µmol/g) = 0.314 · *R2* * − 0.96 with *R2* * = 1000/T2* (4)

There are different grades of hepatic iron overload depending on the concentration of iron in the liver (in µmol/g dry weight). The reference values for the different *LIC*s based on Gandon’s method are [32]: <36 µmol/g (absence of iron overload), 36–80 µmol/g (mild iron overload), and >80 (severe iron overload).

The SIR method in hepatic iron overload [33] was used to compare *S_Liver_* with *S_Paravertebral muscles_* (Equation (2)), as they do not store iron.

## 3. Results

### 3.1. Phantoms

**Figure 2:**
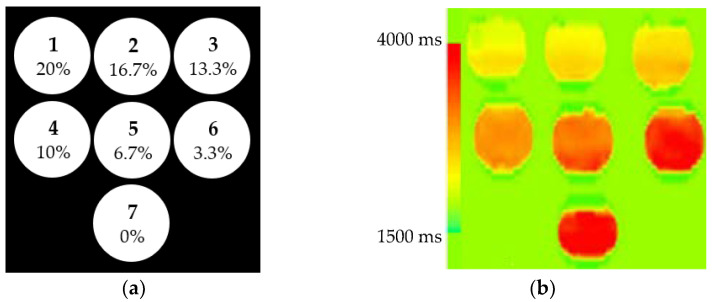
HSA concentrations of the 7 phantoms implemented in this work (**a**) and their *T1* map at 3T (**b**).

The distribution of the HSA concentrations of the 7 phantoms, and the *T1* map of these phantoms, are shown in Figure 2a,b, respectively. From the result, it is clear that the *T1* values increase as the HSA concentration of the phantoms decreases. 

**Figure 3:**
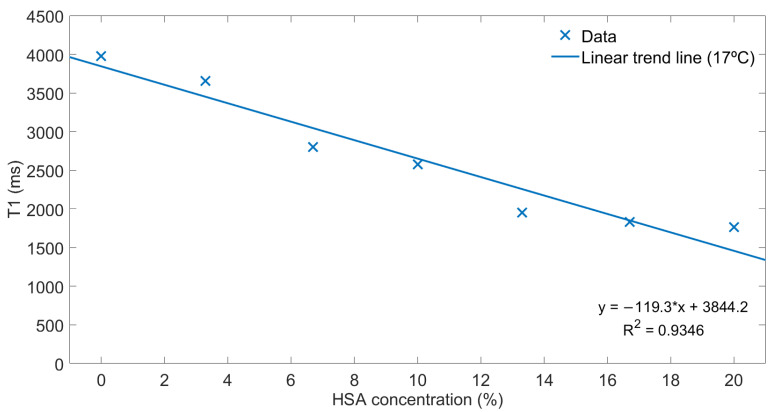
Relationship between *T1* values and HSA concentrations in the 7 phantoms at 3T.

Figure 3 depicts the graphical representation of the *T1* values (ms) as a function of the different HSA concentrations (%). A linear trend line is shown with its respective equation and its coefficient of determination (R^2^ = 0.9346). Additionally, this data set has a Pearson correlation coefficient (r) of −0.9662.

### 3.2. Fibrosis

**Figure 4:**
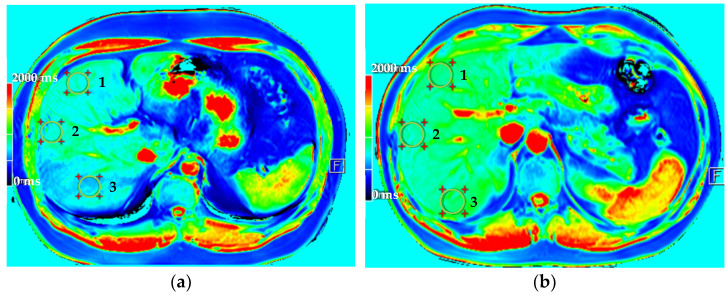
*T1* maps of patient #1 (**a**) and patient #2 (**b**) acquired at 3T. There are 3 ROIs in the liver following Couinaud’s segments IV (1), VIII (2) and VII (3).

The presence of liver fibrosis was evaluated by comparing the *T1* values from the *T1* maps (ms) of a control subject (patient #1) shown in Figure 4a, with a patient with previous evidence of mild fibrosis (patient #2) shown in Figure 4b.

*T1* values for patient #1 are 750.88 ms ± 7.95 ms (mean ± standard deviation): ROI 1, 797.48 ± 66.59 ms; ROI 2, 766.20 ± 49.72 ms; ROI 3, 688.96 ± 66.6 ms. While for patient #2 they are 882.92 ms ± 3.84 ms (mean ± standard deviation): ROI 1, 889.09 ± 44.84 ms; ROI 2, 829.30 ± 50.34 ms; ROI 3, 930.37 ± 54.27 ms.

### 3.3. Steatosis

**Figure 5:**
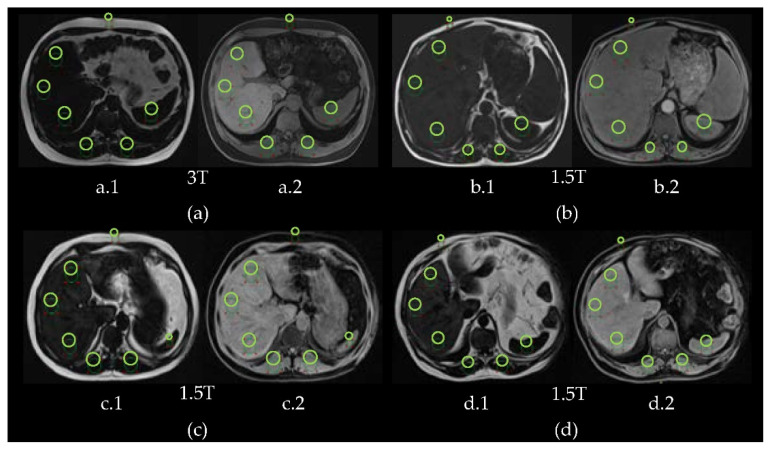
Fat-only images (**a.1**,**b.1**,**c.1**,**d.1**) and water-only images (**a.2**,**b.2**,**c.2**,**d.2**) of patients #3, #4, #5 and #6. In each image there are 3 ROIs in the liver, 2 ROIs in paravertebral muscles, 1 ROI in spleen and 1 ROI in abdominal fat. (**a**) Patient #3 (acquired at 3T): no steatosis (FF = 3.1 ± 1.7%). (**b**) Patient #4 (acquired at 1.5T): mild steatosis (FF = 16.8 ± 1.6%). (**c**) Patient #5 (acquired at 1.5T): moderate steatosis (FF = 17.5 ± 2.4%). (**d**) Patient #6 (acquired at 1.5T): severe steatosis (FF = 24.8 ± 4.6%).

Fat-only images (Figure 5(a.1,b.1,c.1,d.1)) and water-only images (Figure 5 (a.2,b.2,c.2,d.2)) allow the calculation of the FF (%) (Equation (1)) at 3T and 1.5T. The results of the different grades of steatosis show the hyperintensity of the liver when the severity of the disease increases in fat-only images.

**Figure 6:**
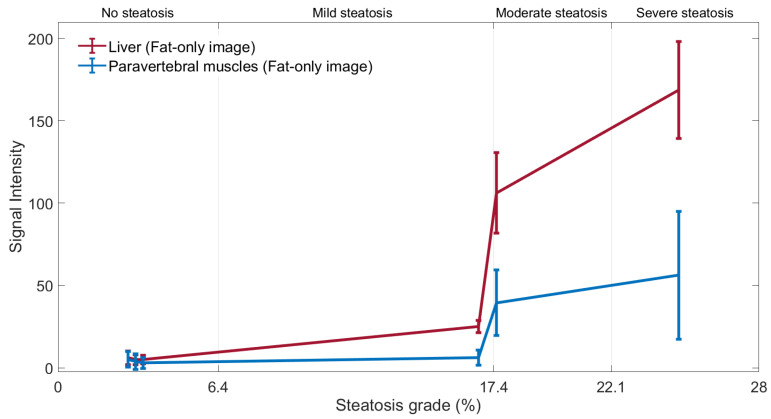
Average signal intensity and standard deviation bars of the ROIs in the liver and the paravertebral muscles in fat-only images for the different grades of steatosis: no steatosis (at 3T), mild steatosis (at 1.5T), moderate steatosis (at 1.5T) and severe steatosis (at 1.5T).

The signal intensity of the liver is compared with the paravertebral muscles signal intensity, which is considered as the reference signal, as it does not change significantly in the presence of steatosis. In these cases, the liver signal is greater than the paravertebral muscles signal for *FF* > 6.4% as it is shown in Figure 6.

### 3.4. Hepatic Iron Overload

**Figure 7:**
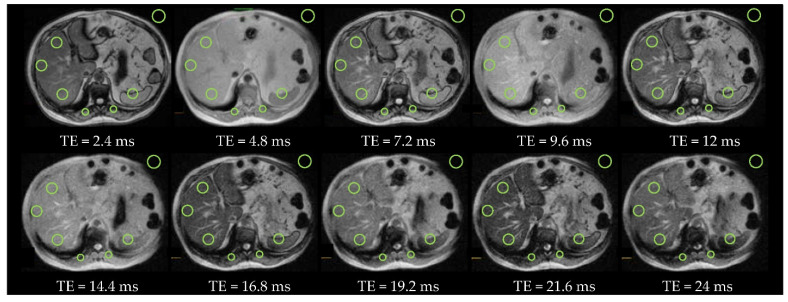
Patient #6 with absence of iron overload and severe steatosis at 1.5T (T2* = 17.5 ms and LIC = 34 µmol/g). There are 10 echoes with a duration of 2.4 ms each echo. The OOP images are with *TE* = 2.4 ms, 7.2 ms, 12 ms, 16.8 ms, and 21.6 ms and IP images are with *TE* = 4.8 ms, 9.6 ms, 14.4 ms, 19.2 ms, and 24 ms. There are 3 ROis in the liver, 2 ROIs in paravertebral muscles, 1 ROI in the spleen and 1 ROI of background noise.

**Figure 8:**
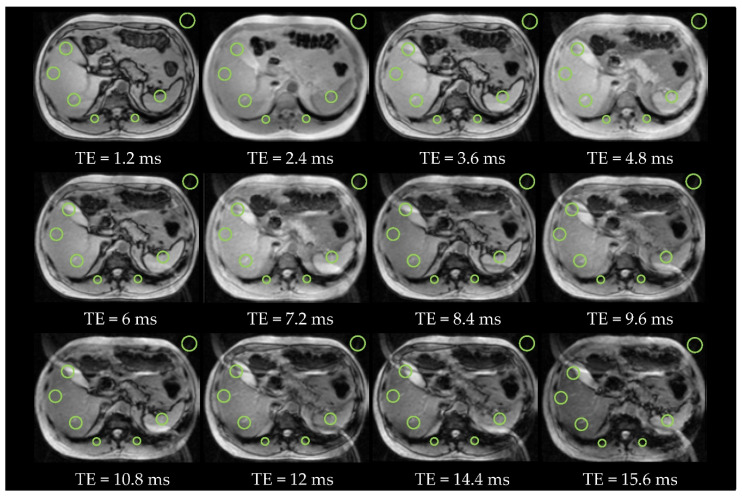
Patient #3 with absence of iron overload and steatosis at 3T (T2* = 16.1 ms and *LIC* = 19 µmol/g). There are 12 echoes with a duration of 1.2 ms each one. The OOP images are with *TE* = 1.2 ms, 3.6 ms, 6 ms, 8.4 ms, 10.8 ms, and 15.6 ms and IP images are with *TE* = 2.4 ms, 4.8 ms, 7.2 ms, 9.6 ms, 12 ms, and 14.4 ms. There are 3 ROis in the liver, 2 ROIs in paravertebral muscles, 1 ROI in the spleen and 1 ROI of background noise.

Figure 7 and Figure 8 present the *T2_Multi-echo* sequence with 10 echoes and 12 echoes in two subjects (patients #6 and #3) with absence of iron overload, acquired at 1.5T and at 3T respectively. 

**Figure 9:**
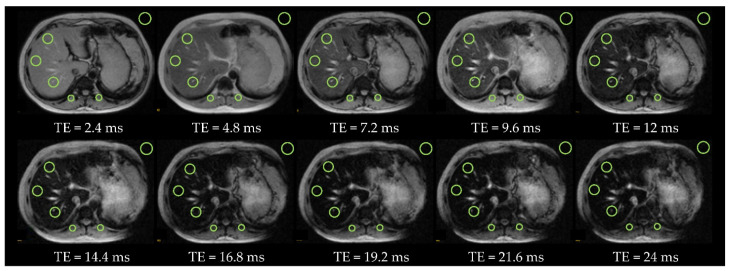
Patient #5 with severe iron overload and moderate steatosis at 1.5T (T2* = 6.2 ms and *LIC* = 95 µmol/g). There are 10 echoes with a duration of 2.4 ms each echo. The OOP images are with *TE* = 2.4 ms, 7.2 ms, 12 ms, 16.8 ms, and 21.6 ms and IP images are with *TE* = 4.8 ms, 9.6 ms, 14.4 ms, 19.2 ms, and 24 ms. There are 3 ROIs in the liver, 2 ROIs in paravertebral muscles, and 1 ROI of background noise.

Figure 9 shows the *T2_Multi-echo* sequence with 10 echoes, acquired at 1.5T, from a subject (patient #5) with severe iron overload.

**Figure 10:**
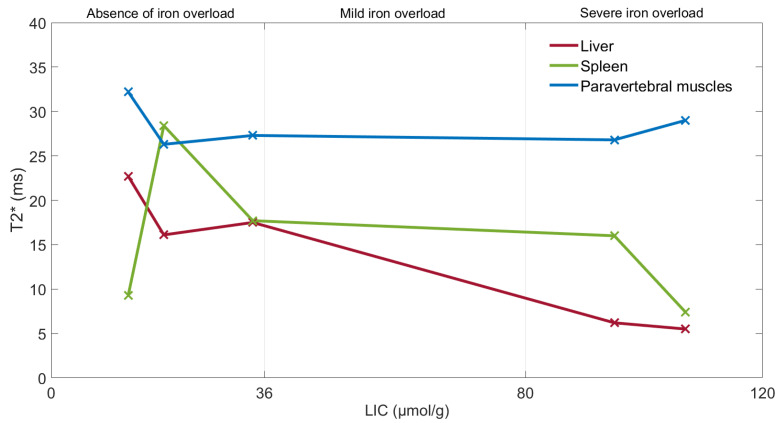
Relationship between the T2* values of the liver, spleen and paravertebral muscles in absence of iron overload at 3T (13 µmol/g and 19 µmol/g) and at 1.5T (34 µmol/g) and severe iron overload at 1.5T (95 µmol/g and 107 µmol/g).

The relationship between the variation of T2* values (ms) in the liver, spleen, and paravertebral muscles, for absence of iron overload (at 3T and 1.5T) and severe iron overload (at 1.5T), is shown in Figure 10. The reference signal is the paravertebral muscles signal because it does not vary significantly when there is hepatic iron overload. 

## 4. Discussion

### 4.1. Fibrosis

The calibration of the *T1* relaxation times with the 7 HSA phantoms showed the inverse relationship (r = −0.9662) between *T1* values and the protein concentration, i.e., the higher concentration of HSA, the lower value of *T1* time. This idea of studying the relationship between the *T1* relaxation time and this protein came from the relationship between the collagen concentration and *T1* maps in liver and cardiac fibrosis [20,25]. Additionally, the selected protein is synthesized exclusively by the liver and some studies, such as the study by Carvalho and Machado [26], established that its accumulation decreases in advanced fibrosis.

The presence of fat and iron has to be taken into account in *T1* maps as they modify the *T1* relaxation times. It is expected that, in advanced fibrosis, the values of the *T1* relaxation times will be higher than those obtained in this patient with previous signs of mild fibrosis. In addition, there is a need to increase the sample size and verify the results with a fibrosis reference method such as elastography.

### 4.2. Steatosis

The *FF* (%) was calculated with fat-only and water-only images which are reconstructed by post-processing IP and OOP images. For this reason, there are not differences in the calculation of the *FF* at 1.5T and 3T, although there is more influence in the decrease in the signal in IP images at 3T if there are steatosis and hepatic iron overload. 

The SIR method is another way to assess hepatic iron overload. The results showed that the liver signal is greater than the paravertebral muscles signal when there is steatosis, and this ratio between both signals increases progressively as the grade of steatosis increases. It is also possible to compare *S_Liver_* with the spleen signal (*S_Spleen_*), but this method loses accuracy if there is iron overload in the liver, in the spleen, or in both organs. In addition, the severity of this disease can be seen through the hyperintensity of the liver in fat-only images in a qualitative manner.

The *T2_Multi-echo* sequence that consists of IP and OOP images can be used to assess steatosis because the signal of the liver decreases in fatty livers in OOP images. The results of the *FF* (%) between *T2_Multi-echo* and *T1_VIBE_Dixon* sequences did not present great differences, although the presence of hepatic iron overload affects the calculation of the *FF* (%) in both sequences. Therefore, the fat calculation can be omitted when there is severe iron overload.

### 4.3. Hepatic Iron Overload

The hepatic iron overload sequence is based on T2-weighted IP and OOP images with multiple echoes, between 10 and 12 echoes, in order to calculate the T2* transverse relaxation time with the exponential relaxation model. The IP images are more sensitive to iron because the *TE* of IP images is greater than the *TE* of OOP images and, as a result of that fact, the liver is hypointense when it has higher amounts of iron.

The duration of the echoes at 1.5T is 2.4 ms, while at 3T it is 1.2 ms because the value of T2* is approximately double at 1.5T than at 3T. Thus, the higher the magnetic field (3T), the shorter the initial *TE* in order to diagnose light iron overloads with greater precision, while severe iron overloads obtain better results with a lower magnetic field (1.5T). In addition, the effect of iron in the liver, and the decay of T2*, is proportional to the magnetic field [32]. 

Gandon’s method showed how the liver signal decreases rapidly, according to the exponential relaxation model, when the hepatic iron overload increases. Furthermore, T2* values of the liver progressively decrease below the reference signal of the paravertebral muscles as the *LIC* (µmol/g) increases.

The offset, or background noise, in steatosis was not relevant, but in iron overload, it can be used as a limit of the liver signal intensity. In other words, if the exponential curve of the liver is less than the background noise, T2* is overestimated, and iron overload is underestimated. Moreover, the signal-to-noise ratio is greater at 3T than at 1.5T, as can be observed in the case of absence of iron overload at 3T and 1.5T.

### 4.4. Limitations and Strengths

The principal limitations of this study are the small sample size of healthy and sick patients and the lack of liver biopsy as a control method. Despite these limitations, it has been possible to start a new pathway to study a non-invasive fibrosis biomarker for clinical routine. Additionally, fibrosis was evaluated when the *T1* relaxation time (ms) increases, and this was displayed by means of the color scale in *T1* maps, while steatosis and hepatic iron overload were evaluated with three methods: (1) Liver fat quantification with *FF* (%), hepatic iron overload quantification with the study of T2* (ms), and its correlation with *LIC* (µmol/g) using Gandon’s method. (2) The SIR method between the liver signal and the paravertebral muscles signal that increased in steatosis and decreased in hepatic iron overload. (3) Hyperintensity of the liver in fat-only images in steatosis and hypointensity of the liver in IP images in comparison with OOP images in hepatic iron overload. Furthermore, it is remarkable the influence of fat and iron in *T1* values, T2* values, and signal intensities, which interfere in the reliable calculation of fibrosis, steatosis, and hepatic iron overload.

Future lines of this study should increase the size and variety of the sample of healthy and sick patients. This will allow them to study and assess the biomarker of fibrosis using the same scanner, as it was calibrated with the phantoms, compare the reference values of the different grades of steatosis in the literature and their possible difference at 1.5T and 3T, study the prevalence of steatosis with anthropometric indicators, such as BMI, abdominal fat signal, and waist index and with other parameters, such as age and gender, study other models for the calculation of T2*, and compare correlations of other authors between T2* (ms) and *LIC* (µmol/g).

## 5. Conclusions

It has been defined as an MRI protocol consisting of four sequences for the assessment of fibrosis, steatosis, and iron overload. The inverse relationship between the *T1* relaxation times and the different HSA concentrations, and its application as a biomarker, showed a promising pathway for the assessment of fibrosis, especially of advanced fibrosis, that future works should investigate with a larger sample of patients and with a fibrosis reference method. The presence of fat (*FF* > 6.4%) and iron overload (36 µmol/g dry weight) was detected with the Dixon method and Gandon’s method, respectively, and with the relationship between the liver signal and paravertebral muscles signal, that increased in steatosis and decreased in hepatic iron overload.

## Figures and Tables

**Table 1:** Patient sample data for fibrosis, steatosis and hepatic iron overload.

Disease		Patient	Age (Years Old)	Gender	Magnetic Field (T) of the MRI Scanner
Fibrosis		#1 *	41	Male	3
		#2	41	Male	3
Steatosis	Hepatic iron overload	#3 **	42	Male	3
		#4	53	Male	1.5
		#5	47	Male	1.5
		#6	79	Male	1.5
		#7	33	Male	3
		#8	24	Female	3

In each disease, a healthy subject is considered as a reference for comparison with other subjects with different levels of the disease. * Control subject for fibrosis. ** Control subject for steatosis and hepatic iron overload.

**Table 2:** Sequence data for the assessment of fibrosis, steatosis and hepatic iron overload at 3T.

Sequences	TR (ms)	TE (ms)	FA (°)	ETL	MS	ST (mm)
*B1Map_for_T1mapping*	5050	1.8	8	1	64 × 52	8
*T1_Images_B1corr*	5	2.3	3	1	224 × 135	4
*T1_VIBE_Dixon*	4	1.3	9	2	320 × 195	3
*T2_Multi-echo*	120	1.2	20	12	128 × 128	7

TR: repetition time; TE: echo time; FA: flip angle; ETL: echo train length; MS: matrix size; ST: slice thickness.

## Data Availability

The datasets generated and analyzed during the current study are available from the corresponding author on reasonable request.
